# Application of the guidewire anchoring technique in lateral approach transforaminal endoscopic lumbar foraminoplasty: a propensity score–matched retrospective study

**DOI:** 10.3389/fsurg.2026.1893563

**Published:** 2026-08-27

**Authors:** Guoxing Ding, Huijing Shang, Fuhai Yan, Weijie Cui, Zhi Li, Yiheng Zhang, Zhan Wang, Yifan Feng, Junxiao Su, Hui Zhang

**Affiliations:** 1 The First Clinical Medical College of Gansu University of Traditional Chinese Medicine, Lanzhou, Gansu, China; 2 Department of Pain Medicine, Liangzhou Hospital, Wuwei, Gansu, China; 3 Department of Spine Surgery, Gansu Provincial People’s Hospital, Lanzhou, Gansu, China; 4 College of Basic Medicine, Mudanjiang Medical University, Heilongjiang, China; 5 Department of Orthopedics, The First Affiliated Hospital of Tsinghua University, Beijing, China

**Keywords:** anchoring guidewire technique, foraminoplasty, lumbar disc herniation, percutaneous endoscopic lumbar discectomy, superior articular process

## Abstract

**Objective:**

Safe and rapid bony localization for intervertebral foraminoplasty is essential in lateral transforaminal endoscopic lumbar discectomy (TELD). This study introduces a novel guidewire anchoring puncture technique and compares it with conventional puncture to evaluate operative efficiency, safety, and clinical outcomes.

**Methods:**

A retrospective 1:1 propensity score-matched (PSM) analysis was performed on 60 patients with single-level L4/5 or L5/S1 disc herniation receiving TELD (anchoring group = 30, conventional group = 30). Baseline demographics, intraoperative metrics, neurological complications, and serial Oswestry Disability Index (ODI)/leg pain Numeric Rating Scale (NRS) scores (preoperatively, 1-day, 3-month, 12-month postoperatively) were compared. *Post-hoc* power calculation and linear mixed-effects models were applied for statistical validation.

**Results:**

Baseline characteristics were balanced after PSM (all standardized mean differences < 0.1). The anchoring group exhibited significantly shorter total operative time, superior articular process (SAP) foraminoplasty time, and fewer fluoroscopic exposures (all *P* < 0.001). Transient nerve irritation occurred in 3.3% (1/30) of anchoring patients versus 20.0% (6/30) of conventional patients; only one nerve injury (3.3%) appeared in the conventional group (Fisher's exact test *P* values provided). Intergroup NRS/ODI differences were non-significant at preoperative, 1-day, and 3-month timepoints (*P* > 0.05). Although the anchoring group had statistically lower 12-month NRS scores (*P* = 0.0027), the between-group difference failed to meet the established minimal clinically important difference (MCID) for leg pain.

**Conclusion:**

The guidewire anchoring technique improves TELD foraminoplasty efficiency and reduces intraoperative radiation and neurological complication risk. However, given the single-center, small-sample, 12-month limited follow-up design, its broad clinical adoption requires further multicenter prospective validation. Both techniques deliver equivalent clinically meaningful long-term pain relief.

## Introduction

Minimally invasive spinal endoscopy has become mainstream for lumbar disc herniation, preserving spinal anatomy while achieving decompression comparable to open surgery. Two classic lateral/posterior endoscopic approaches exist: transforaminal endoscopic lumbar discectomy (TELD) and interlaminar endoscopic lumbar discectomy (IELD) (1). Two classic TELD systems dominate clinical practice: the Yeung Endoscopic Spine System (YESS) and Hoogland's TESSYS technique (2). YESS shows limitations for high-migrated, sequestered herniations combined with spinal stenosis. The TESSYS system addresses this gap via intentional SAP foraminoplasty: ventral resection of the superior articular process enlarges the foramen and creates a safe working corridor for endoscope placement (3). Sufficient foraminoplasty directly reduces nerve root injury risk and enables complete disc fragment removal (4). Traditional foraminoplasty carries notable drawbacks. Soft tissue obstruction, unstable cannula positioning for novice surgeons, and variable bony hyperplasia hinder rapid SAP tip identification (5). These challenges trigger repeated fluoroscopic verification, prolonged operative duration, and increased patient discomfort under local anesthesia. Inadequate bony resection also leaves residual bone fragments that cause persistent radicular pain (6).

Multiple modified puncture and localization strategies have been proposed to optimize TELD workflow in recent years, including CT real-time navigation and robot-assisted transforaminal endoscopy. Such navigation-assisted protocols improve puncture accuracy but substantially raise equipment costs and extend preoperative preparation time, limiting widespread routine use in primary hospitals (7, 8). Our guidewire anchoring modification requires no additional expensive navigation hardware and delivers consistent trajectory guidance with minimal fluoroscopy demand, offering a cost-effective alternative to robot/CT-guided techniques. We developed a guidewire anchoring puncture method to streamline SAP localization: the wire anchors at the posterolateral vertebral body margin adjacent to the disc, providing a fixed trajectory for sequential dilation and targeted bony resection. Preliminary clinical use demonstrated reduced radiation exposure and shorter surgery time. This retrospective PSM study systematically compares intraoperative metrics, complication profiles, and serial clinical outcomes between the anchoring technique and standard conventional puncture TELD.

## Materials and methods

### Study population & eligibility

A retrospective review of patients with single-level L4/5 or L5/S1 disc herniation undergoing TELD at the Spine Surgery Department of Gansu Provincial People’s Hospital (January 2022–December 2023) was conducted.

Inclusion criteria:

1. Radiating leg pain with positive straight-leg raise test; leg pain predominating over low back pain.
2. CT/MRI confirmation of single-level protruded or sequestered disc herniation.
3. Persistent symptoms after ≥3 months standardized conservative management.

Exclusion criteria:

1. Spinal canal calcification/ossification.
2. Lumbar instability, spondylolisthesis, severe degenerative scoliosis.
3. Prior same-segment spinal surgery or recurrent disc herniation.
4. Spinal tumor, tuberculosis, or active spinal infection.

Ethics approval was obtained from the institutional Ethics Committee (No. 2025-329). All patients provided written informed consent, and the study complied with the Declaration of Helsinki.

All surgeries were completed by a fixed surgical team with ≥100 independent standard TELD cases before implementing the novel anchoring technique. All operators fully completed the standard TELD learning curve before applying the anchoring protocol; learning curve bias on operative time and fluoroscopy dosage was therefore minimized.

**Figure 1:**
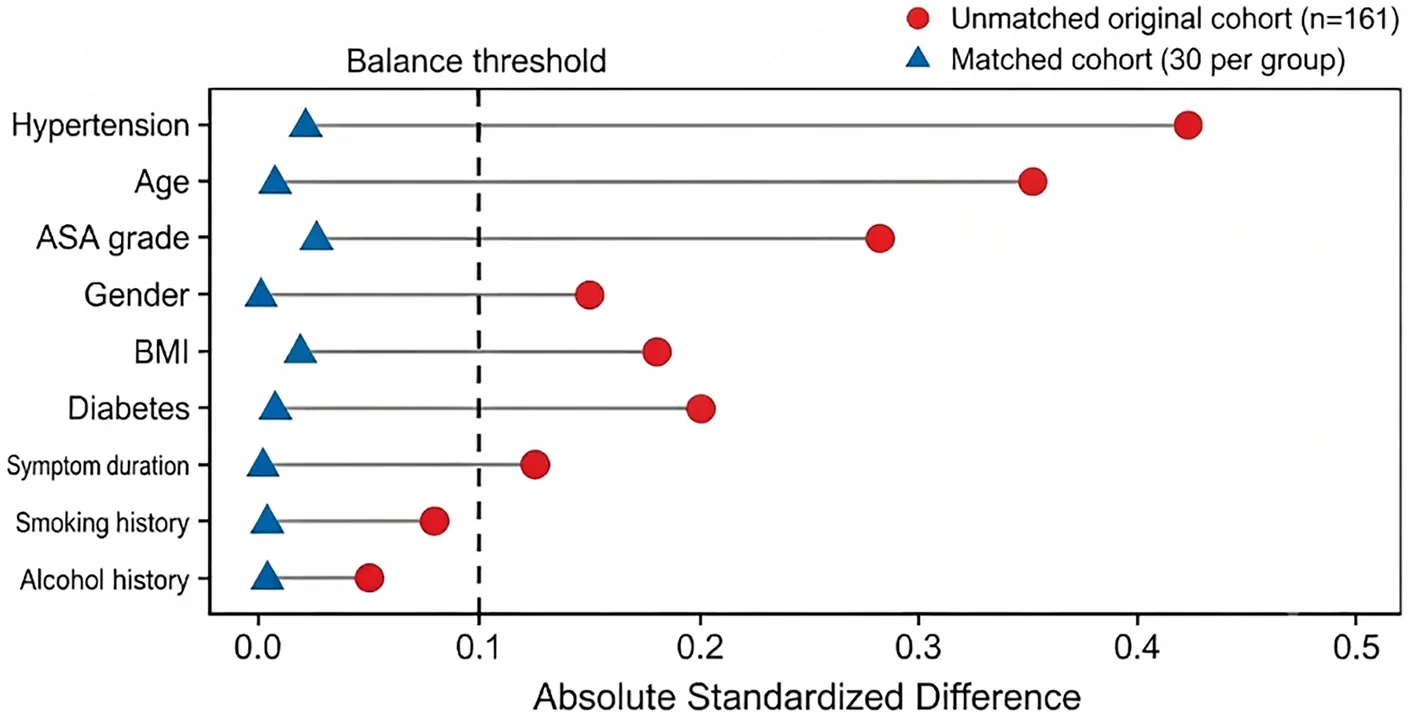
Love plot of absolute standardized mean differences for 9 baseline covariates before and after 1:1 nearest-neighbor propensity score matching.

**Figure 2:**
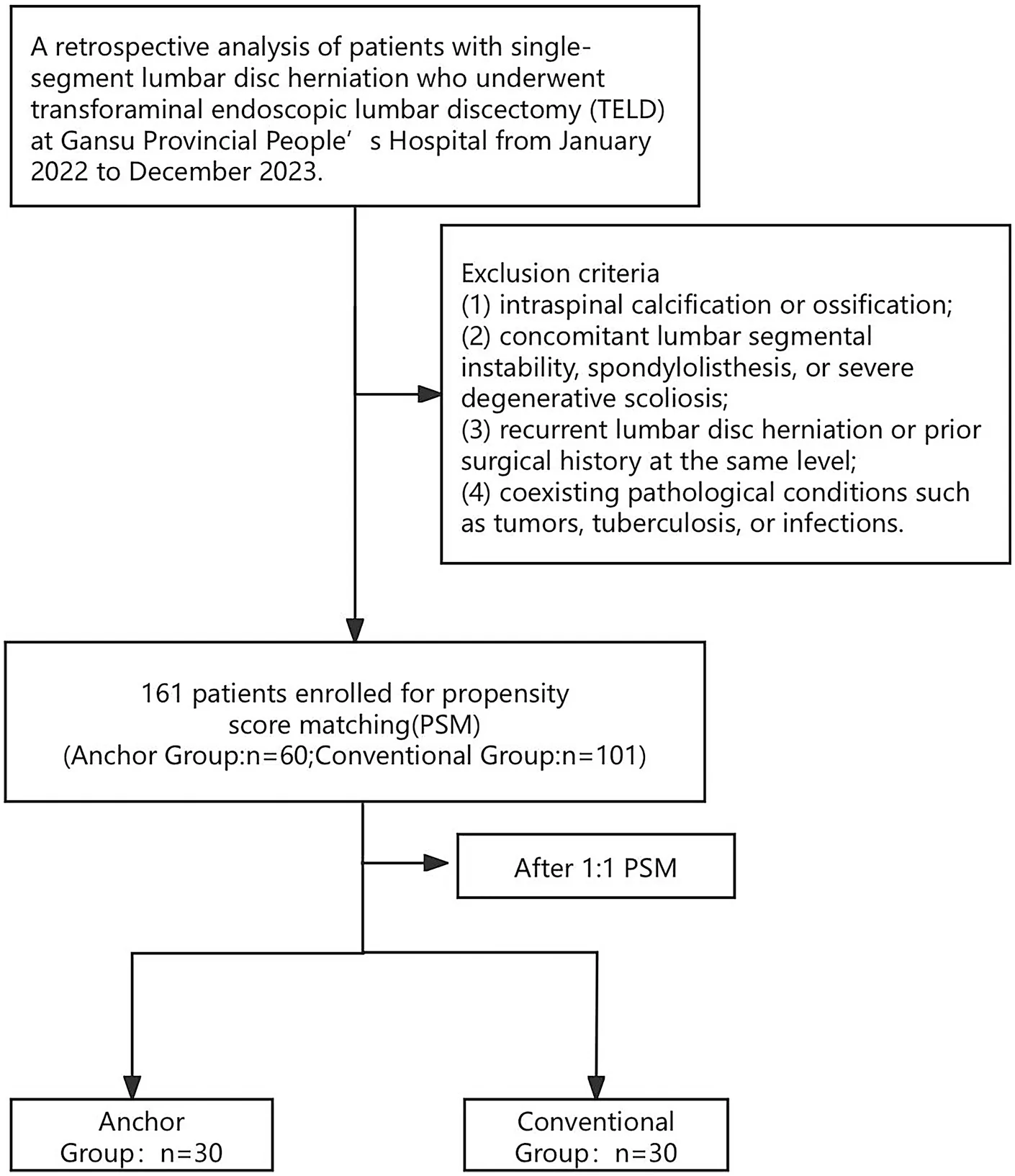
Flowchart of patient screening, exclusion, and 1:1 propensity score matching.

PSM was applied to mitigate selection bias from non-random group allocation and improve baseline comparability between groups. A 1:1 nearest-neighbor matching algorithm without replacement was adopted, with a caliper width set to 0.02. Matching covariates included age, sex, BMI, ASA grade, symptom duration, smoking history, alcohol use, hypertension, and diabetes. Baseline balance was evaluated via standardized mean differences (SMD), with |SMD| < 0.1 defined as satisfactory intergroup balance per widely recognized criteria. A total of 161 initially eligible patients were screened, and 30 patients were retained in each group after matching. The exact SMD values of all covariates are presented in Table 1, and a Love plot was generated to visually display the balance changes before and after matching (Figure 1). A flowchart illustrating patient screening, exclusion and matching procedures is shown in Figure 2.

**Table 1:** Absolute standardized mean differences of baseline covariates before and after 1:1 propensity score matching.

Covariate	Unmatched SMD	Matched SMD
Hypertension	0.351	0.018
ASA grade	0.412	0.059
Age	0.284	0.035
Symptom duration	0.32	0.078
Gender	0.215	0.012
Alcohol history	0.203	0.021
BMI	0.176	0.027
Diabetes	0.148	0.045
Smoking history	0.115	0.039

|SMD| < 0.1 indicates acceptable baseline balance between groups. Matching adopted 1:1 nearest-neighbor matching without replacement (caliper width = 0.02).

## Surgical protocols

All patients received local anesthesia (1% ropivacaine 10 mL + 2% lidocaine 10 mL + 0.9% normal saline 10 mL) and were positioned prone.

### Anchoring technique group

**Figure 3:**
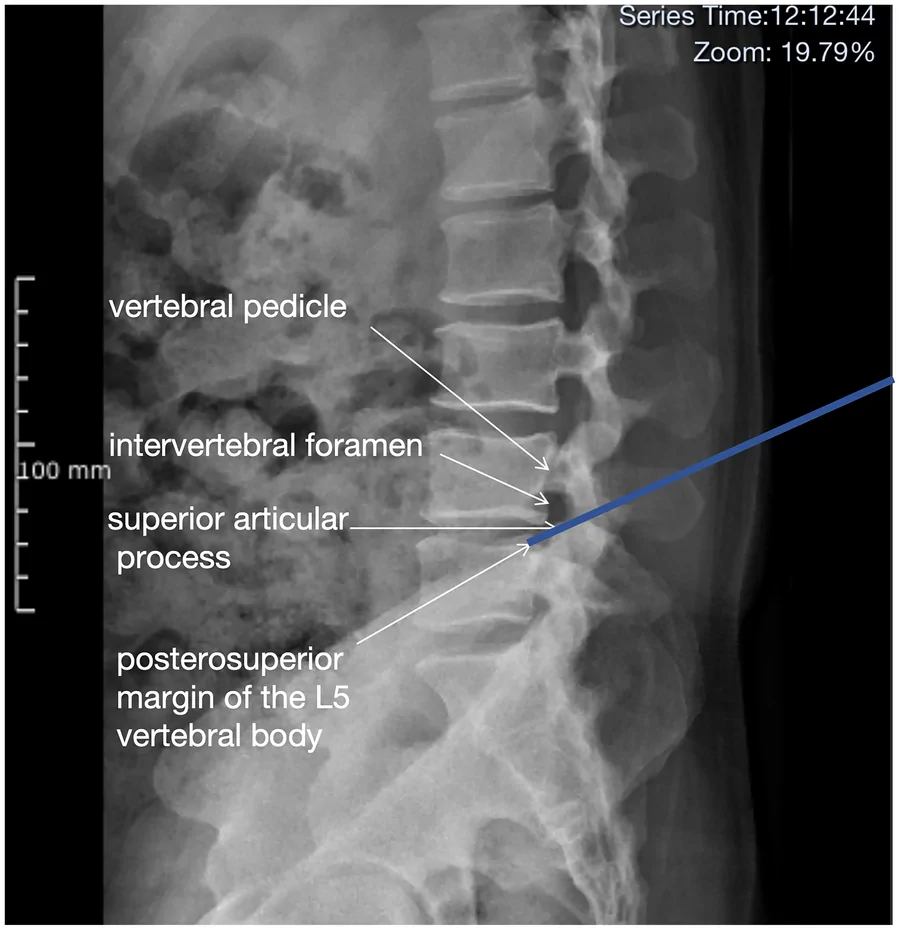
Fluoroscopic schematic showing the blue anchoring guidewire passing the L5 SAP tip to the posterosuperior L5 vertebral body corner.

**Figure 4:**
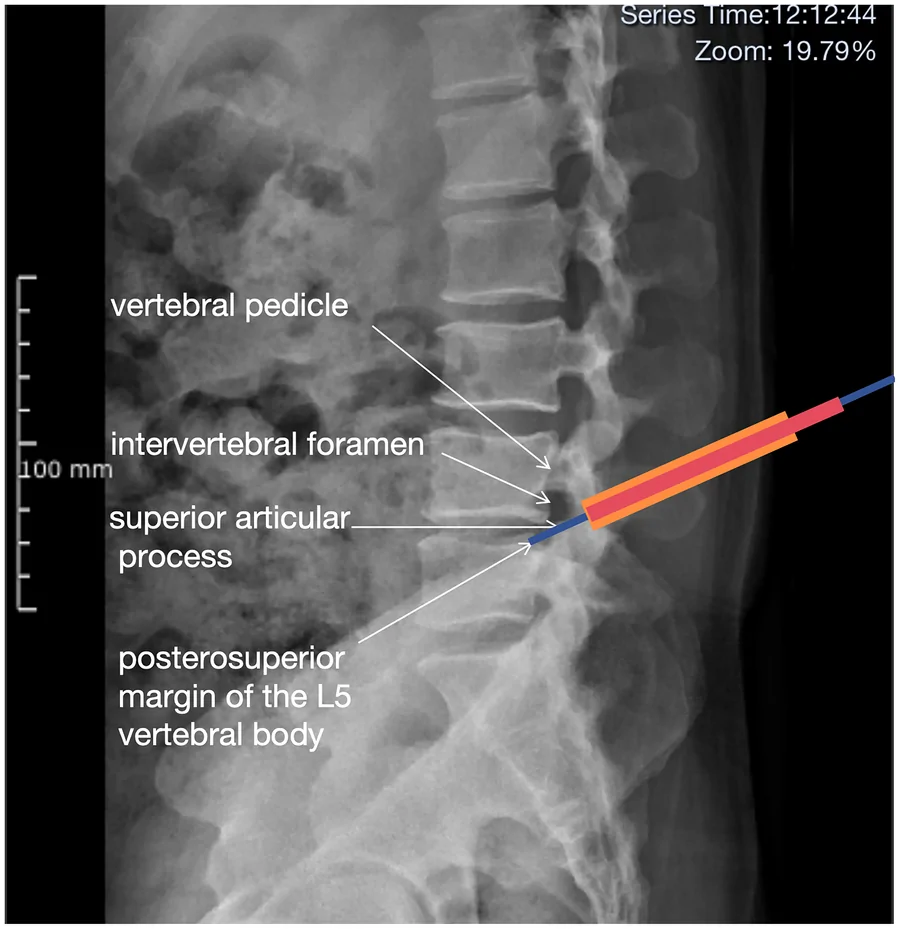
Yellow-labeled U-shaped working cannula placed along the fixed guidewire.

**Figure 5:**
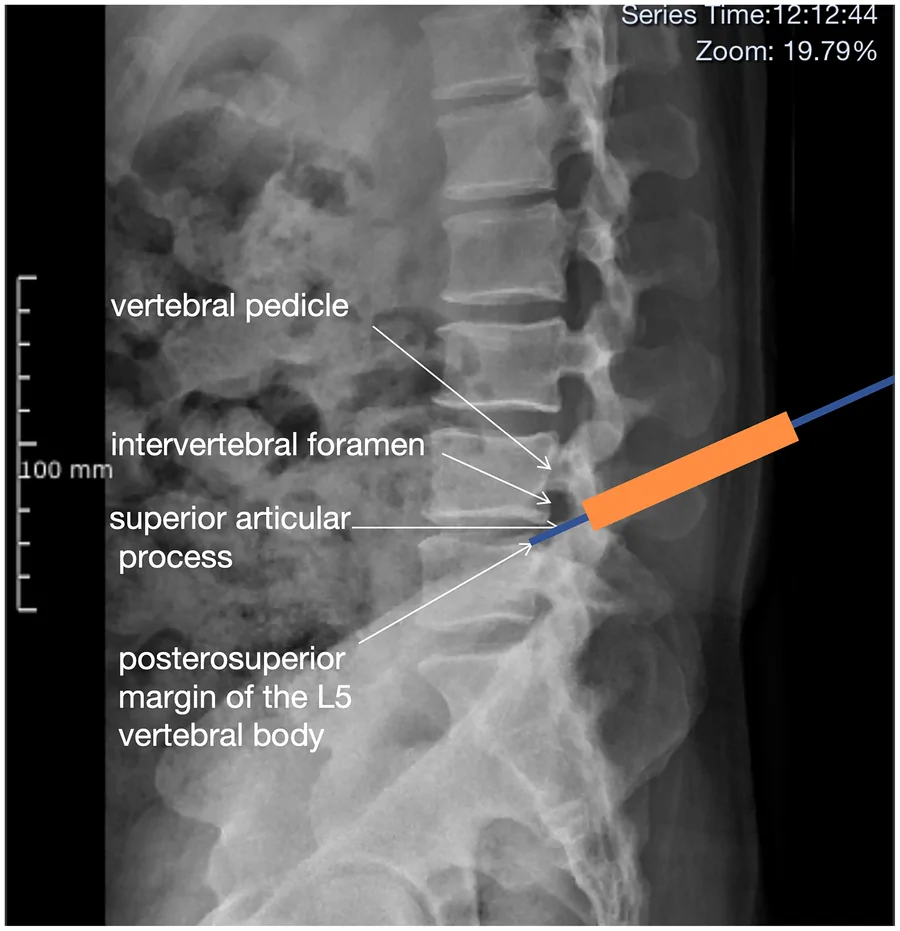
Endoscopic system advanced through the wire-guided corridor for SAP visualization.

Preoperative fluoroscopy marked the target puncture trajectory. An 18G needle advanced toward the SAP tip, with layered local anesthetic infiltration. Upon bony contact, additional 8 mL anesthetic was injected. Under biplanar fluoroscopy, the needle crossed the SAP tip and terminated at the posterolateral margin of the inferior vertebral body; a guidewire was secured at this anchoring position, confirmed via radiography. After needle removal, an 8 mm skin incision was made, and sequential dilators were advanced along the fixed guidewire to form a preliminary working corridor. Blunt dissection cleared soft tissue overlying the SAP, followed by insertion of a U-shaped cannula. Dilators were removed while retaining the anchoring wire, then a T-shaped cannula was delivered over the wire. The endoscope tracked the guidewire to clearly visualize the SAP complex. Radiofrequency probes performed soft tissue dissection and hemostasis. The T-cannula was temporarily withdrawn, and an external circular saw tracked along the wire to resect hyperplastic ventral SAP bone for foraminoplasty. After bony decompression, the saw was removed, the T-cannula reinserted and rotated to expose the target disc, and the guidewire was extracted before discectomy. Representative fluoroscopic illustrations of guidewire and cannula placement are shown in Figures 3–5.

### Conventional puncture group

Standard fluoroscopy-guided puncture targeted the SAP tip without fixed guidewire anchoring. After wire placement and sequential dilation, U-shaped cannula insertion was performed; dilators and the temporary guidewire were removed immediately. A T-shaped cannula and endoscope were inserted, and SAP localization depended on repeated cannula rotation and radiofrequency dissection before circular saw foraminoplasty.

Universal intraoperative steps for both groups: endoscopic discectomy and nerve root decompression, confirmation of free dural/nerve root pulsation to terminate surgery. No postoperative drainage tubes or routine antibiotics were administered. All patients wore a lumbar brace for 2 months and initiated ambulatory functional training on postoperative day 1.

## Outcome assessments

1. Operative time, superior articular process foraminoplasty time (from working cannula insertion to completion of foraminoplasty), and number of effective fluoroscopy exposures were recorded and compared.
2. Clinical Outcomes: Functional status and pain were evaluated preoperatively and at 1 day, 3 months, and 1 year postoperatively using the Oswestry Disability Index (ODI) and the numeric rating scale (NRS) for leg pain (9, 10). Surgical efficacy at the final follow-up was assessed using the MacNab criteria.
3. Complications: Postoperative complications were recorded, including nerve irritation symptoms (paresthesia, electric shock–like pain, localized numbness, or sensory deficits) and nerve injury (primarily motor weakness (11).
4. The Minimal Clinically Important Difference (MCID) threshold for 0–10 leg pain NRS in lumbar spinal surgery was predefined as 1–2 points based on published consensus standards (12). Any intergroup NRS difference below this cutoff lacks tangible clinical significance, even if statistically significant.
5. All clinical outcomes were assessed by independent investigators based on standardized criteria. Due to the retrospective nature of this study, blinding of outcome assessors to patient grouping was not feasible.

## Statistical analysis

Statistical analyses were performed using SPSS version 22.0 (IBM Corp., Armonk, NY, USA). Propensity score matching (PSM), covariate balance assessment, and Love plot generation were performed using R version 4.2.1 (R Foundation for Statistical Computing, Vienna, Austria). PSM was conducted using 1:1 nearest-neighbor matching with a caliper width of 0.2 standard deviations of the logit of the propensity score estimated by logistic regression. Covariate balance was assessed using standardized mean differences (SMDs), with an absolute SMD < 0.1 indicating adequate balance. Normality and homogeneity of variance were assessed using the Shapiro–Wilk and Levene's tests, respectively. Continuous variables were compared using the Mann–Whitney U test, whereas categorical variables were analyzed using the *χ*^2^ test or Fisher's exact test, as appropriate. Within-group comparisons were performed using paired t-tests or Wilcoxon signed-rank tests according to data distribution. All tests were two-sided, and *P* < 0.05 was considered statistically significant.

## Results

### Comparison of baseline characteristics between the two groups

The initial review involved a total of 161 eligible patients; however, only 60 were ultimately included in the PSM analysis. The anchoring group consisted of 30 patients (mean age, 42.5 ± 10.1 years), and the conventional group also included 30 patients (mean age, 44.8 ± 9.7 years). The anchoring group comprised 17 males and 13 females, with a mean BMI of 24.5 ± 3.4 kg/m^2^. In the group of participants who consumed the conventional diet, there were 19 males and 11 females included in the study, with a mean BMI of 24.7 ± 3.6 kg/m^2^. The present study revealed that there were no significant differences observed between the two groups in terms of sex, age, BMI, disease duration, operative level distribution, smoking history, alcohol consumption, hypertension history or ASA classification (*P* > 0.05). This indicates good comparability between the groups (Table 2).

**Table 2:** Demographic and clinical characteristics of the two patient groups after propensity score matching.

	Anchor group (*N* = 30)	Conventional group (*N* = 30)	*t*/*χ*2 value	*P*
Age (yr)	42.5 ± 10.1	44.8 ± 9.7	−0.898	0.373
Sex (women/men)	17/13	11/19	2.410	0.120
BMI (kg/m2)	24.5 ± 3.4	24.7 ± 3.6	−0.224	0.823
Symptom duration (mo)	4.1 ± 1.5	3.9 ± 1.6	0.499	0.620
Operative level, *n* (%)			0.268	0.605
L4–L5	15 (50)	17 (57)		
L5–S1	15 (50)	13 (43)		
Smoking history (yes/no)	1/29	2/28		1.000
Alcohol consumption history (yes/no)	1/29	1/29		1.000
History of diabetes (yes/no)	0/30	3/27		0.239
History of hypertension (yes/no)	3/27	2/28		1.000
ASA grade, *n* (%)			0.434	0.805
I	18 (60)	16 (53.3)		
II	10 (33.3)	11 (36.7)		
III	2 (6.7)	3(10)		

ASA, American Society of Anesthesiologists; BMI, body mass index.

### Comparison of surgical parameters and complications

Compared with the conventional puncture group, the anchoring group demonstrated significantly shorter operative time, superior articular process foraminoplasty time, and fewer effective fluoroscopy exposures (all *P* < 0.001). Regarding complications, transient nerve irritation occurred in 1 patient (3.3%) in the anchoring group, compared with 6 patients (20.0%) in the conventional group, showing a downward trend. One patient (3.3%) in the conventional group experienced nerve injury, whereas no such cases occurred in the anchoring group (Table 3).

**Table 3:** Presents a comparison of the surgical parameters and complications exhibited by the two cohorts.

	Anchor group (*N* = 30)	Conventional group (*N* = 30)	*z*/*χ*2 value	*P*
TOT(min)	45.8 ± 5.2	65.3 ± 6.8	−12.48	0.000
SAPPT (min)	12.5 ± 2.1	20.7 ± 3.4	−11.24	0.000
NEF (time)	8.6 ± 1.9	18.2 ± 3.1	−14.46	0.000
NS (%)	1 (3.3)	6 (20.0)	–	0.056
NI (%)	0 (0.0)	1 (3.3)	–	1.000

TOT, total operative time; SAPPT, superior articular process plasty time; NEF, number of effective fluoroscopies; NS, neurogenic symptoms; NI, nerve injury.

### Comparison of clinical outcomes

Follow-up results showed that both groups experienced significant improvements in leg pain NRS scores and ODI scores at all postoperative time points compared with preoperative values (*P* < 0.001). No significant differences were found between the two groups in preoperative, 1-day postoperative, or 3-month postoperative NRS scores, or in ODI scores at any time point (*P* > 0.05), suggesting that both approaches achieved satisfactory clinical decompression. As demonstrated in Figure 1, the NRS scores at the 12-month postoperative stage were found to be significantly reduced in the anchor group when compared to the conventional group (*P* < 0.05) (Table 4).

**Table 4:** The following investigation will compare the ODI and NRS scores of the two groups prior to and following surgery.

	Anchor group (*N* = 30)	Conventional group (*N* = 30)	*p*-value^†^	*t*-value
NRS for leg pain				
Preoperative	7.2 ± 0.9	7.4 ± 1.0	0.419	−0.814
Postoperative (1 day)	2.1 ± 0.8*	2.4 ± 1.1*	0.232	−1.208
Postoperative (3 months)	1.0 ± 0.5*	1.2 ± 0.7*	0.208	−1.274
Postoperative (12 months)	0.93 ± 0.06*	1.033 ± 0.17*	0.0027	−3.131
ODI scores				
Preoperative	70.5 ± 8.2	71.6 ± 7.9	0.599	−0.529
Postoperative (1 day)	25.1 ± 5.6*	26.8 ± 6.0*	0.262	−1.134
Postoperative (3 months)	14.3 ± 4.1*	15.5 ± 4.8*	0.302	−1.041
Postoperative (12 months)	11.67 ± 1.25*	12.45 ± 2.13*	0.089	−1.729

* Compared with preoperative, *p* < 0.001.

† Comparison between the two groups.

## Discussion

Clinical Importance of Adequate SAP Foraminoplasty Early “inside-out” TELD techniques limited visualization of the traversing/exiting nerve roots, risking persistent radicular irritation for patients with sequestered discs or spinal stenosis (13). Targeted SAP foraminoplasty expands the narrow lumbar foramen to create a safe, spacious corridor for endoscope delivery and complete epidural decompression (14–16). Novice surgeons face consistent difficulty identifying bony SAP landmarks without stable trajectory guidance, leading to prolonged surgery and excessive fluoroscopy radiation relative to experienced operators (17–19).

Mechanisms and Core Advantages of the Guidewire Anchoring Technique. The anchoring wire is locked at the disc-vertebral body junction to form a fixed intraoperative trajectory, delivering five key improvements over free-hand conventional puncture: 1. Stable bony landmark localization eliminates interference from soft tissue swelling or facet hyperplasia. 2. Minimizes repeated fluoroscopic checks to reduce patient and surgeon radiation exposure. 3. Supports smooth sequential dilation to stabilize the working channel and reduce intraoperative bleeding. 4. Avoids repeated cannula manipulation near nerve roots, lowering transient radicular irritation risk. 5. Streamlines surgical workflow to shorten total operative and foraminoplasty time.

Comparative Intraoperative and Clinical Outcomes Consistent with our primary hypothesis, the anchoring group achieved shorter surgery times and fewer fluoroscopic shots, alongside a lower rate of transient nerve irritation. Prior modified puncture instruments similarly reduce radiation burden but rely on specialized proprietary hardware unavailable to most primary care hospitals (5, 20). Both techniques generated equivalent clinically meaningful pain and functional recovery at early and mid-term follow-up. While the anchoring group showed statistically superior 12-month NRS values, the tiny between-group difference did not reach the MCID cutoff, confirming equivalent long-term clinical efficacy from a patient-centered perspective. This critical distinction between statistical and clinical significance is emphasized to avoid overinterpretation of minor numerical differences.

### Safety interpretation limitation

In this study, transient nerve irritation occurred in 3.3% of the anchoring group versus 20.0% of the conventional group, while permanent nerve injury was only observed in one patient (3.3%) receiving standard puncture. Excessive repeated cannula manipulation and traction on traversing/exiting nerve roots have been identified as the primary cause of postoperative sensory and motor dysfunction in routine TELD, which can be effectively avoided via the fixed anchoring trajectory. The wire-stabilized working corridor restricts uncontrolled sliding of the circular burr during bony foraminoplasty and lowers the risk of residual bone fragments, a well-documented trigger for persistent postoperative radiculopathy (21). Reported neurological complication rates across large TELD retrospective cohorts range from 0% to 12.4% (22–24), which aligns with the adverse event profile observed in our cohort. Although the anchoring group exhibited numerically fewer neurological adverse events, the absolute number of complications was extremely limited (7 nerve irritation events and 1 permanent nerve injury among 60 participants). Such a low event count limits our ability to draw definitive statistical conclusions regarding superior neurological safety of the anchoring technique; larger cohorts with extended follow-up are required to verify its long-term protective effect on neural structures.

### Study limitations

1. Retrospective single-center design: All procedures performed by one fixed experienced surgical team. Results cannot be generalized to other hospitals or less experienced operators, limiting external validity. Outcome evaluators were unblinded to group assignment, introducing potential assessment bias.
2. Small sample size (*N* = 60 total patients): *post-hoc* power analysis revealed limited statistical power to detect small differences in rare adverse neurological events. The short 12-month follow-up window cannot evaluate long-term disc recurrence or late degenerative complications; published literature supports 12-month endpoints for short-term recovery but cannot substitute multi-year follow-up.
3. Missing perioperative secondary endpoints: Medical record data did not capture intraoperative blood loss, hospitalization duration, reoperation rates, or time to return to daily activity, preventing full multidimensional comparison of technique recovery profiles.
4. PSM cannot eliminate all unmeasured residual confounding inherent to non-randomized retrospective research.
5. All operators had completed the standard TELD learning curve before using the anchoring wire; outcomes may differ for early-career surgeons still mastering basic transforaminal puncture.

## Conclusion

The guidewire anchoring puncture technique optimizes TELD foraminoplasty workflow by shortening operative time and minimizing fluoroscopic radiation exposure, with a numerically lower incidence of transient neurological irritation relative to conventional free-hand puncture. However, definitive conclusions regarding universal safety superiority are constrained by the small sample size and low adverse event count. Both surgical approaches yield comparable clinically meaningful pain and functional improvement through 12 months of follow-up. Given the single-center, retrospective study limitations, the anchoring technique cannot be recommended for broad universal adoption without further multicenter prospective validation. This low-cost, navigation-free modified puncture method offers incremental intraoperative efficiency benefits suitable for experienced spine surgeons performing routine TELD procedures.

## Data Availability

The original contributions presented in the study are included in the article/Supplementary Material, further inquiries can be directed to the corresponding author.
